# Supplementary material for: Comparative Analysis on the Evolution of Flowering Genes in Sugar Pathway in Brassicaceae
Source: Genes (Basel). 2022 Sep 28;13(10):1749. doi: 10.3390/genes13101749 (PMC9602146; doi:10.3390/genes13101749)
Supplement: Supplementary file 1 [file genes-13-01749-s001.zip › genes-1895131-supplementary.pdf]

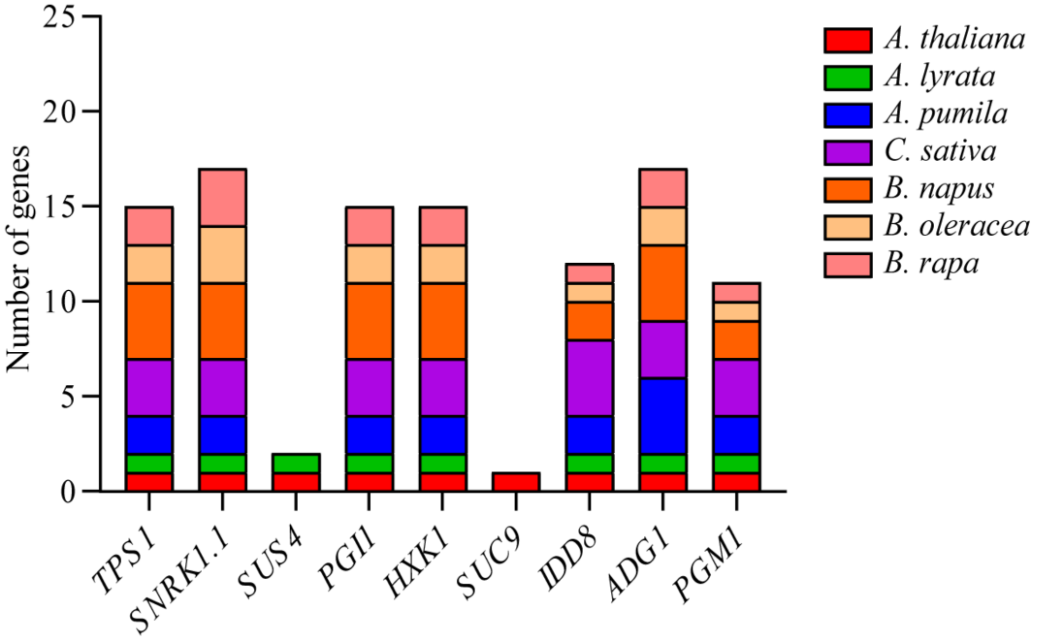

Figure S1 The number of the identified sugar pathway gene members associated with flowering from seven Brassicaceae species

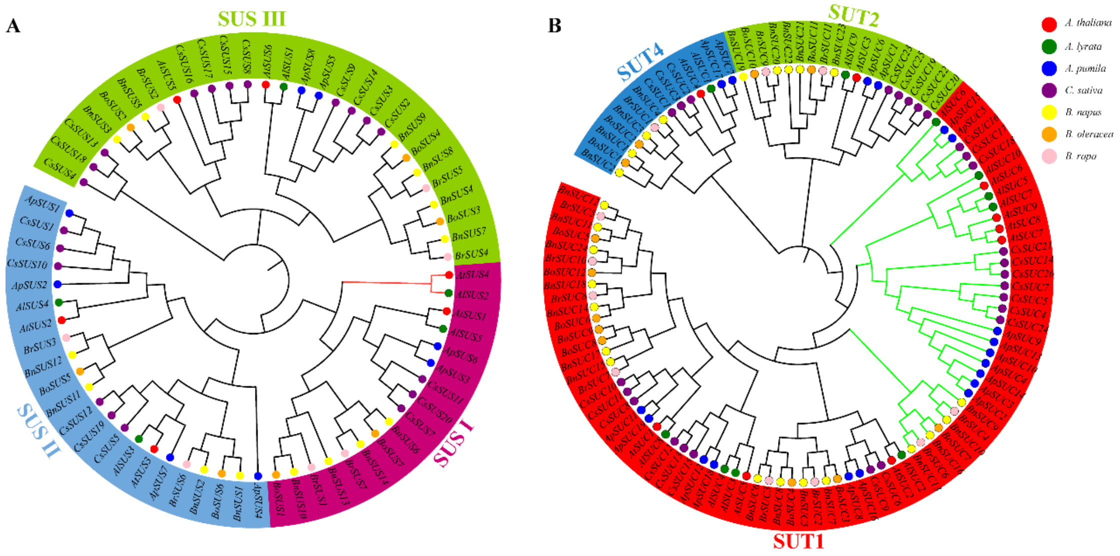

Figure S2 Phylogenetic trees of SUS and SUC families from seven cruciferous plant. A: Phylogenetic trees of SUS gene family; B: Phylogenetic trees of SUC gene family. The red line indicates the genetic evolution branches of atsus4 and six other plants, The green line indicates the genetic evolution branches of *AtSUC9* and six other plants, details of the original names corresponding to the names of members of SUS and SUC gene families in table S4 and table S5.

Table S1 Genes related to flowering transition in the sugar pathway in *Arabidopsis thaliana*

Abbreviation of gene	Full name of gene	Gene ID	References
TPS1	*TREHALOSE-6-PHOSPHATE SYNTHASE 1*	AT1G78580	Wahl et al, 2013
AKIN10, *SNRK1.1*	*SNF1 KINASE HOMOLOG 10*	AT3G01090	Jeong et al, 2015
SUS4	*SUCROSE SYNTHASE 4*	AT3G43190	Baroja-Fernández et al, 2012
PGI1	*PHOSPHOGLUCOSE ISOMERASE 1*	AT4G24620	Yu et al, 2000
HXK1, *GIN2*	*HEXOKINASE 1*	AT4G29130	Yang et al, 2013
SUC9	*SUCROSE-PROTON SYMPORTER 9*	AT5G06170	Sivitz et al., 2007
IDD8, *NUC*	*INDETERMINATE DOMAIN 8*	AT5G44160	Seo et al., 2011
ADG1, *APS1*	*ADP GLUCOSE PYROPHOSPHORYLASE 1*	AT5G48300	Yu et al, 2000
PGM1	*PHOSPHOGLUCOMUTASE*	AT5G51820	Yu et al, 2000

Table S2 Names of SUS gene family members in seven Brassicaceae species

Species	Gene	Gene ID
Arabidopsis thaliana	*AtSUS1*	AT5G20830.1
	AtSUS2	AT5G49190.1
	AtSUS3	AT4G02280.1
	AtSUS4	AT3G43190.1
	AtSUS5	AT5G37180.1
	AtSUS6	AT1G73370.1
Arabidopsis lyrata	*AlSUS1*	fgenesh1_pm.C_scaffold_2001317
	AlSUS2	fgenesh2_kg.5__780__AT3G43190.1
	AlSUS3	fgenesh2_kg.6__3476__AT4G02280.1
	AlSUS4	fgenesh2_kg.8__796__AT5G49190.1
	AlSUS5	scaffold_602179.1
Arabidopsis pumila	*ApSUS1*	evm.model.9__7_CONTIGS_16776222.209
	ApSUS2	evm.model.12__9_CONTIGS_14700693.1833
	ApSUS3	evm.model.4__12_CONTIGS_18100624.2820
	ApSUS4	evm.model.4__12_CONTIGS_18100624.738.1
	ApSUS5	evm.model.11__11_CONTIGS_16209776.1538
	ApSUS6	evm.model.6__5_CONTIGS_17268225.1633
	ApSUS7	evm.model.6__5_CONTIGS_17268225.236.1
	ApSUS8	evm.model.14__14_CONTIGS_14340626.772
Camelina sativa	*CsSUS1*	Csa11g078470.1
	CsSUS2	Csa16g036080.1
	CsSUS3	Csa16g036080.2
	CsSUS4	Csa03g006230.1
	CsSUS5	Csa08g052190.1
	CsSUS6	Csa18g018540.1
	CsSUS7	Csa08g014600.1
	CsSUS8	Csa10g040720.1
	CsSUS9	Csa09g077930.1
	CsSUS10	Csa02g035280.1
	CsSUS11	Csa20g032780.1
	CsSUS12	Csa02g005510.1
	CsSUS13	Csa20g016510.1
	CsSUS14	Csa07g040560.1
	CsSUS15	Csa11g050910.1
	CsSUS16	Csa12g074560.2
	CsSUS17	Csa12g074560.1
	CsSUS18	Csa20g016510.2
	CsSUS19	Csa13g054950.1
	CsSUS20	Csa13g024710.1
	BnSUS1	GSBRNA2T00007838001
	BnSUS2	GSBRNA2T00018414001
	BnSUS3	GSBRNA2T00036780001

Brassica napus	*BnSUS4*	GSBRNA2T00049086001
	BnSUS5	GSBRNA2T00066235001
	BnSUS6	GSBRNA2T00094365001
	BnSUS7	GSBRNA2T00099500001
	BnSUS8	GSBRNA2T00102645001
	BnSUS9	GSBRNA2T00103416001
	BnSUS10	GSBRNA2T00140859001
	BnSUS11	GSBRNA2T00143079001
	BnSUS12	GSBRNA2T00144354001
	BnSUS13	GSBRNA2T00148916001
	BnSUS14	GSBRNA2T00154198001
Brassica oleracea	*BoSUS1*	BolC03g010920.2J
	BoSUS2	BolC06g017370.2J
	BoSUS3	BolC06g032260.2J
	BoSUS4	BolC06g044870.2J
	BoSUS5	BolC07g038200.2J
	BoSUS6	BolC09g001700.2J
	BoSUS7	BolC09g051640.2J
Brassica rapa	*BrSUS1*	BraA03g009830.3C
	BrSUS2	BraA05g015010.3C
	BrSUS3	BraA06g034940.3C
	BrSUS4	BraA07g028740.3C
	BrSUS5	BraA07g037520.3C
	BrSUS6	BraA09g001880.3C
	BrSUS7	BraA10g019840.3C

Table S3 Names of members of the SUC gene family members in seven *Brassicaceae* species

Species	Gene	Gene ID
Arabidopsis thaliana	*AtSUC1*	AT1G71880
	AtSUC2	AT1G22710
	AtSUC3	AT2G02860
	AtSUC4	AT1G09960
	AtSUC5	AT1G71890
	AtSUC6	AT5G43610
	AtSUC7	AT1G66570
	AtSUC8	AT2G14670
	AtSUC9	AT5G06170
Arabidopsis lyrata	*AlSUC1*	fgenesh2_kg.1__1061__AT1G09960.1
	AlSUC2	fgenesh2_kg.1__2467__AT1G22710.1
	AlSUC3	fgenesh2_kg.2__1445__AT1G71890.1
	AlSUC4	fgenesh2_kg.106__1__AT1G71890.1
	AlSUC5	fgenesh2_kg.6__554__AT1G66570.1
	AlSUC6	scaffold_603288.1
	AlSUC7	Al_scaffold_0002_1232
	AlSUC8	scaffold_202108.1
	AlSUC9	fgenesh2_kg.5__187__AT2G02860.1
	AlSUC10	fgenesh1_pm.C_scaffold_3002472
Arabidopsis pumila	*ApSUC1*	evm.model.13__8_CONTIGS_14517794.145
	ApSUC2	evm.model.4__12_CONTIGS_18100624.3403
	ApSUC3	evm.model.4__12_CONTIGS_18100624.1444
	ApSUC4	evm.model.4__12_CONTIGS_18100624.1470
	ApSUC5	evm.model.4__12_CONTIGS_18100624.219
	ApSUC6	evm.model.3__5_CONTIGS_18323972.2753
	ApSUC7	evm.model.2__6_CONTIGS_19393823.2691
	ApSUC8	evm.model.2__6_CONTIGS_19393823.1520
	ApSUC9	evm.model.7__7_CONTIGS_17117682.171
	ApSUC10	evm.model.7__7_CONTIGS_17117682.2745
	ApSUC11	evm.model.11__11_CONTIGS_16209776.1689
	ApSUC12	evm.model.11__11_CONTIGS_16209776.1688
	ApSUC13	evm.model.6__5_CONTIGS_17268225.2998
	ApSUC14	evm.model.6__5_CONTIGS_17268225.3022
	ApSUC15	evm.model.6__5_CONTIGS_17268225.747
	ApSUC16	evm.model.1__5_CONTIGS_22361435.2083
	ApSUC17	evm.model.1__5_CONTIGS_22361435.899
	ApSUC18	evm.model.14__14_CONTIGS_14340626.915
	ApSUC19	evm.model.14__14_CONTIGS_14340626.916
	CsSUC1	Csa03g013400.1
	CsSUC2	Csa14g011470.1
	CsSUC3	Csa17g013590.1

	CsSUC4	Csa04g024680.1
	CsSUC5	Csa04g024680.2
	CsSUC6	Csa17g029640.1
	CsSUC7	Csa09g035840.1
	CsSUC8	Csa09g075220.1
	CsSUC9	Csa14g027910.1
	CsSUC10	Csa07g038820.1
	CsSUC11	Csa16g034520.1
	CsSUC12	Csa07g038830.1
Camelina sativa	*CsSUC13*	Csa16g034540.1
	CsSUC14	Csa20g008390.1
	CsSUC15	Csa09g075230.1
	CsSUC16	Csa02g028390.1
	CsSUC17	Csa13g043590.1
	CsSUC18	Csa08g037440.1
	CsSUC19	Csa09g005070.1
	CsSUC20	Csa04g008350.2
	CsSUC21	Csa08g058350.1
	CsSUC22	Csa04g008350.1
	CsSUC23	Csa06g002850.1
	CsSUC24	Csa06g018450.1
	CsSUC25	Csa09g005070.2
	CsSUC26	Csa20g008380.1
	BnSUC1	GSBRNA2T00037540001
	BnSUC2	GSBRNA2T00156127001
	BnSUC3	GSBRNA2T00019276001
	BnSUC4	GSBRNA2T00011511001
	BnSUC5	GSBRNA2T00033135001
	BnSUC6	GSBRNA2T00015756001
	BnSUC7	GSBRNA2T00061853001
	BnSUC8	GSBRNA2T00119488001
	BnSUC9	GSBRNA2T00134421001
	BnSUC10	GSBRNA2T00049405001
	BnSUC11	GSBRNA2T00103236001
Brassica napus	*BnSUC12*	GSBRNA2T00102508001
	BnSUC13	GSBRNA2T00029602001
	BnSUC14	GSBRNA2T00137296001
	BnSUC15	GSBRNA2T00099581001
	BnSUC16	GSBRNA2T00107013001
	BnSUC17	GSBRNA2T00062437001
	BnSUC18	GSBRNA2T00116464001
	BnSUC19	GSBRNA2T00073348001
	BnSUC20	GSBRNA2T00030810001
	BnSUC21	GSBRNA2T00115350001

	BnSUC22	GSBRNA2T00062975001
	BnSUC23	GSBRNA2T00081619001
	BnSUC24	GSBRNA2T00081760001
Brassica oleracea	*BoSUC1*	BolC05g007810.2J
	BoSUC2	BolC08g056300.2J
	BoSUC3	BolC07g020620.2J
	BoSUC4	BolC05g021040.2J
	BoSUC5	BolC06g043310.2J
	BoSUC6	BolC02g028540.2J
	BoSUC7	BolC09g065780.2J
	BoSUC8	BolC09g022600.2J
	BoSUC9	BolC06g033450.2J
	BoSUC10	BolC02g050720.2J
	BoSUC11	BolC07g032500.2J
	BoSUC12	BolC07g057680.2J
Brassica rapa	*BrSUC1*	BraA09g062110.3C
	BrSUC2	BraA07g014520.3C
	BrSUC3	BraA09g040330.3C
	BrSUC4	BraA03g002390.3C
	BrSUC5	BraA07g036350.3C
	BrSUC6	BraA10g028880.3C
	BrSUC7	BraA07g029350.3C
	BrSUC8	BraA02g020540.3C
	BrSUC9	BraA02g035840.3C
	BrSUC10	BraA03g058820.3C
	BrSUC11	BraA06g039570.3C

Table S4 Accession numbers of RNA-Seq data of *Arabidopsis pumila* nine different tissues

Accession number	Library ID	Tissues
SRR14470720	EM	Embryo
SRR14470722	YR	Young root
SRR14470717	MR	Mature root
SRR14470714	RL	Rosette leaf
SRR14470712	CL	Cauline leaf
SRR14470713	BU	Bud
SRR14470719	FL	Flower
SRR14470724	SL	Silique
SRR14470710	SE	Seed

Table S5 Primer sequences used in qPCR assay

Primer	Primer sequence (5'→3')
ApADG1-1-F	GACATCTTTCCCGTGCGTAT
ApADG1-1-R	AGTCTCCCTATGCGCTTGAA
ApADG1-2-F	AAGGCCATGAAGGTTGACAC
ApADG1-2-R	CTTCGCTTCCGAAGTCATTC
ApADG1-4-F	GCTGATGCTGTGAGGCAGTA
ApADG1-4-R	AGGAAGAGCAGCAACCGTAA
ApGADPH-F	CTCCCATGTTTGTTGTTGGTGTCA
ApGADPH-R	CTTCCACCTCTCCAGTCCTTCATT

Table S6 Information on the identified sugar pathway gene members identified to be associated with flowering from seven Brassicaceae species

Species	Gene	Gene ID	Chromosome No.	Amino acid No.	Molecular weight (kDa)	PI	Subcellular localization
	AtTPS1	AT1G78580	Chr1	942	105.98	6.70	Cell wall and Vacuole
	AtSNRK1.1	AT3G01090	Chr3	535	61.18	8.44	Nucleus
	AtSUS4	AT3G43190	Chr3	808	93.00	6.12	Chloroplast
Arabidopsis thaliana	*AtPGI1*	AT4G24620	Chr4	613	67.05	5.47	Cytoplasm
	AtHXK1	AT4G29130	Chr4	496	53.71	5.76	Chloroplast and Mitochondrion
	AtSUC9	AT5G06170	Chr5	491	52.58	9.13	Cell membrane
	AtIDD8	AT5G44160	Chr5	466	51.19	8.36	Nucleus
	AtADG1	AT5G48300	Chr5	520	56.65	6.13	Chloroplast
	AtPGM1	AT5G51820	Chr5	623	67.99	5.48	Chloroplast
	AlTPS1	fgenesh2_kg.2__2152__AT1G78580.1	Chr2	955	107.40	6.92	Cell wall; Cytoplasm and Vacuole
	AlSNRK1.1	fgenesh1_pm.C_scaffold_3000097	Chr3	509	58.05	8.01	Nucleus
	AlSUS4	fgenesh2_kg.5__780__AT3G43190.1	Chr5	808	92.64	6.02	Chloroplast
Arabidopsis lyrata	*AlPGI1*	fgenesh2_kg.7__1814__AT4G24620.1	Chr7	613	67.13	5.42	Cytoplasm
	AlHXK1	scaffold_701340.1	Chr7	496	53.74	5.66	Chloroplast and Mitochondrion
	AlIDD8	scaffold_800604.1	Chr8	464	51.17	8.61	Nucleus
	AlADG1	fgenesh2_kg.8__698__AT5G48300.1	Chr8	521	57.06	6.30	Chloroplast
	AlPGM1	fgenesh2_kg.8__1063__AT5G51820.1	Chr8	618	67.31	5.32	Chloroplast
	ApTPS1-1	evm.model.14__14_CONTIGS_14340626.258	Chr14	944	106.24	6.80	Cell wall and Vacuole
	ApTPS1-2	evm.model.11__11_CONTIGS_16209776.830	Chr9	947	106.59	6.81	Cell wall; Cytoplasm and Vacuole
	ApSNRK1.1-1	evm.model.5__10_CONTIGS_17087769.3140	Chr7	397	45.83	6.29	Nucleus
	ApSNRK1.1-2	evm.model.8__3_CONTIGS_16920646.3113	Chr8	392	45.19	5.93	Nucleus
	ApPGI1-1	evm.model.0__8_CONTIGS_29314649.4500	Chr5	613	67.29	5.43	Cytoplasm
	ApPGI1-2	evm.model.0__8_CONTIGS_29314649.1181	Chr11	614	67.46	5.49	Cytoplasm
Arabidopsis pumila	*ApHXK1-1*	evm.model.0__8_CONTIGS_29314649.4954	Chr5	498	53.89	5.71	Chloroplast and Mitochondrion

	ApHXK1-2	evm.model.0__8_CONTIGS_29314649.1623	Chr11	498	54.03	5.80	Chloroplast and Mitochondrion
	ApIDD8-1	evm.model.12__9_CONTIGS_14700693.2307	Chr15	465	50.76	8.46	Nucleus
	ApIDD8-2	evm.model.9__7_CONTIGS_16776222.2421	Chr10	460	50.27	8.44	Nucleus
	ApADG1-1	evm.model.12__9_CONTIGS_14700693.1936	Chr15	510	55.71	5.92	Chloroplast
	ApADG1-2	evm.model.9__7_CONTIGS_16776222.114	Chr10	511	55.97	5.80	Chloroplast
	ApADG1-3	evm.model.5__10_CONTIGS_17087769.582	Chr7	518	56.76	6.30	Chloroplast
	ApADG1-4	evm.model.8__3_CONTIGS_16920646.847	Chr8	518	56.79	6.11	Chloroplast
	ApPGM1-1	evm.model.9__7_CONTIGS_16776222.478	Chr10	626	68.17	5.40	Chloroplast
	ApPGM1-2	evm.model.12__9_CONTIGS_14700693.1587	Chr15	626	68.18	5.55	Chloroplast
	CsTPS1-1	Csa09g092680.1	Chr9	1038	117.18	6.95	Vacuole
	CsTPS1-2	Csa07g057580.1	Chr7	1010	113.44	6.51	Cell wall; Cytoplasm and Vacuole
	CsTPS1-3	Csa16g048140.1	Chr16	1095	122.76	8.07	Cell wall and Vacuole
	CsSNRK1.1-1	Csa19g002550.1	Chr19	509	57.98	8.01	Nucleus
	CsSNRK1.1-2	Csa15g001490.1	Chr15	509	57.97	8.01	Nucleus
	CsSNRK1.1-3	Csa01g001460.1	Chr1	509	58.01	8.01	Nucleus
	CsPGI1-1	Csa12g029700.1	Chr12	617	67.62	5.41	Cytoplasm
	CsPGI1-2	Csa10g018420.1	Chr10	615	67.32	5.30	Cytoplasm
	CsPGI1-3	Csa11g020110.1	Chr11	616	67.43	5.37	Cytoplasm
Camelina sativa	*CsHXK1-1*	Csa12g020330.1	Chr12	496	53.65	5.86	Chloroplast and Mitochondrion
	CsHXK1-2	Csa10g013440.1	Chr10	499	54.02	5.77	Chloroplast and Mitochondrion
	CsHXK1-3	Csa11g014380.1	Chr11	499	54.05	5.77	Chloroplast and Mitochondrion
	CsIDD8-1	Csa20g071190.1	Chr20	464	50.68	8.58	Nucleus
	CsIDD8-2	Csa18g009620.1	Chr18	468	51.27	8.45	Nucleus
	CsIDD8-3	Csa20g071190.2	Chr20	453	49.38	8.52	Nucleus
	CsIDD8-4	Csa11g070530.1	Chr11	391	42.20	6.38	Nucleus
	CsADG1-1	Csa18g014380.1	Chr18	518	56.65	5.87	Chloroplast

	CsADG1-2	Csa11g074430.1	Chr11	520	56.83	5.87	Chloroplast
	CsADG1-3	Csa20g041200.1	Chr20	523	57.22	5.87	Chloroplast
	CsPGM1-1	Csa18g022680.1	Chr18	619	67.24	5.38	Chloroplast
	CsPGM1-2	Csa02g044730.1	Chr2	617	66.90	5.09	Chloroplast
	CsPGM1-3	Csa11g083410.1	Chr11	617	66.87	5.13	Chloroplast
	BnTPS1-1	GSBRNA2T00148002001	ChrC06	951	107.08	6.63	Cell wall; Cytoplasm and Vacuole
	BnTPS1-2	GSBRNA2T00146430001	ChrA07	950	106.94	6.63	Cell wall; Cytoplasm and Vacuole
	BnTPS1-3	GSBRNA2T00084348001	ChrC02	932	104.80	6.74	Cell wall; Cytoplasm and Vacuole
	BnTPS1-4	GSBRNA2T00036543001	ChrA02	774	87.35	6.63	Cytoplasm and Vacuole
	BnSNRK1.1-1	GSBRNA2T00017357001	ChrCnn	511	58.04	8.01	Nucleus
	BnSNRK1.1-2	GSBRNA2T00057213001	ChrA01	511	58.08	8.01	Nucleus
	BnSNRK1.1-3	GSBRNA2T00064166001	ChrA05	506	57.63	8.03	Nucleus
	BnSNRK1.1-4	GSBRNA2T00076301001	ChrC05	506	57.67	8.03	Nucleus
	BnPGI1-1	GSBRNA2T00116085001	ChrC01	615	66.98	5.23	Cytoplasm
	BnPGI1-2	GSBRNA2T00048977001	ChrA03	613	66.84	5.38	Cytoplasm
	BnPGI1-3	GSBRNA2T00056331001	ChrA01	616	67.02	5.22	Cytoplasm
Brassica napus	*BnPGI1-4*	GSBRNA2T00154458001	ChrC07	614	66.88	5.23	Cytoplasm
	BnHXK1-1	GSBRNA2T00059441001	ChrA07	500	54.03	5.49	Chloroplast and Mitochondrion
	BnHXK1-2	GSBRNA2T00138056001	ChrC07	499	53.93	5.63	Chloroplast and Mitochondrion
	BnHXK1-3	GSBRNA2T00091626001	ChrA09	499	53.90	5.40	Chloroplast and Mitochondrion
	BnHXK1-4	GSBRNA2T00115923001	ChrC09	499	54.04	5.27	Chloroplast and Mitochondrion
	BnIDD8-1	GSBRNA2T00018774001	ChrA06	464	50.61	8.60	Nucleus
	BnIDD8-2	GSBRNA2T00158200001	ChrC07	464	50.63	8.69	Nucleus
	BnADG1-1	GSBRNA2T00076549001	ChrCnn	511	56.14	6.30	Chloroplast
	BnADG1-2	GSBRNA2T00144434001	ChrA06	513	56.12	6.73	Chloroplast
	BnADG1-3	GSBRNA2T00110776001	ChrCnn	520	57.07	5.99	Chloroplast

	BnADG1-4	GSBRNA2T00038740001	ChrA07	520	57.04	5.86	Chloroplast
	BnPGM1-1	GSBRNA2T00110218001	ChrA10	635	69.12	5.77	Chloroplast
	BnPGM1-2	GSBRNA2T00042494001	ChrCnn	629	68.52	5.48	Chloroplast
Brassica oleracea	*BoTPS1-1*	BolC06g049480.2J	Chr6	951	107.08	6.63	Cell wall; Cytoplasm and Vacuole
	BoTPS1-2	BolC02g034630.2J	Chr2	947	106.42	6.87	Cell wall; Cytoplasm and Vacuole
	BoSNRK1.1-1	BolC01g057210.2J	Chr1	511	58.04	8.01	Nucleus
	BoSNRK1.1-2	BolC05g062460.2J	Chr5	513	58.35	8.00	Nucleus
	BoSNRK1.1-3	BolC03g035940.2J	Chr3	528	59.97	8.79	Nucleus
	BoPGI1-1	BolC01g019030.2J	Chr1	592	64.42	5.05	Cytoplasm
	BoPGI1-2	BolC01g019040.2J	Chr1	518	56.52	5.43	Cytoplasm
	BoHXK1-1	BolC07g001520.2J	Chr7	499	53.92	5.63	Chloroplast and Mitochondrion
	BoHXK1-2	BolC09g014060.2J	Chr9	499	54.04	5.27	Chloroplast and Mitochondrion
	BoIDD8	BolC07g025980.2J	Chr7	464	50.60	8.69	Nucleus
	BoADG1-1	BolC07g037440.2J	Chr7	511	56.07	6.14	Chloroplast
	BoADG1-2	BolC07g010610.2J	Chr7	627	69.16	6.51	Chloroplast
	BoPGM1	BolC09g038330.2J	Chr9	629	68.52	5.48	Chloroplast
Brassica rapa	*BrTPS1-1*	BraA07g041670.3C	Chr9	975	109.93	6.65	Vacuole
	BrTPS1-2	BraA02g024760.3C	Chr2	921	103.67	6.75	Cell wall and Vacuole
	BrSNRK1.1-1	BraA01g044590.3C	Chr1	511	58.06	8.01	Nucleus
	BrSNRK1.1-2	BraA05g040920.3C	Chr5	506	57.63	8.03	Nucleus
	BrSNRK1.1-3	BraA03g030660.3C	Chr3	523	59.642	8.86	Nucleus
	BrPGI1-1	BraA01g015390.3C	Chr1	616	67.08	5.32	Cytoplasm
	BrPGI1-2	BraA03g052000.3C	Chr3	613	66.79	5.33	Cytoplasm
	BrHXK1-1	BraA07g000960.3C	Chr7	500	54.06	5.42	Chloroplast and Mitochondrion
	BrHXK1-2	BraA09g012100.3C	Chr9	499	53.93	5.40	Chloroplast and Mitochondrion
	BrIDD8	BraA06g043270.3C	Chr6	453	49.34	8.76	Nucleus

BrADG1-1	BraA07g007620.3C	Chr7	520	57.04	5.86	Chloroplast
BrADG1-2	BraA06g035670.3C	Chr6	513	56.32	7.09	Chloroplast
BrPGM1	BraA10g010420.3C	Chr10	670	73.34	5.80	Chloroplast

Table S7 The non-synonymous (*K*a) and synonymous (*K*s) of orthologous gene pairs of the sugar pathway members in seven Brassicaceae spp.

Collinear gene pair	Protein sequence similarity (%)	*K*a	*K*s	*K*a/*K*s	Evolutionary selection
AtTPS1 / *AlTPS1*	96.957	0.008360514	0.163302835	0.051196379	Purification selection
AtSNRK1.1 / *AlSNRK1.1*	98.242	0.005539404	0.089613416	0.061814456	Purification selection
AtSUS4 / *AlSUS4*	96.287	0.017094757	0.214461180	0.079710263	Purification selection
AtPGI1 / *AlPGI1*	98.695	0.005781072	0.104807563	0.055158917	Purification selection
AtHXK1 / *AlHXK1*	98.185	0.008011946	0.091830529	0.087247081	Purification selection
AtIDD8 / *AlIDD8*	94.433	0.019881252	0.164226135	0.121060223	Purification selection
AtADG1 / *AlADG1*	95.202	0.023799080	0.19262735	0.123549851	Purification selection
AtPGM1 / *AlPGM1*	94.409	0.016342081	0.160534265	0.101798085	Purification selection
AtTPS1 / *ApTPS1-1*	98.095	0.008949432	0.221002578	0.040494696	Purification selection
AtTPS1 / *ApTPS1-2*	97.996	0.007428389	0.201750621	0.036819659	Purification selection
AtSNRK1.1 / *ApSNRK1.1-1*	97.906	0.045932229	0.190464354	0.241159190	Purification selection
AtSNRK1.1 / *ApSNRK1.1-2*	96.859	0.023642620	0.139939467	0.168948905	Purification selection
AtPGI1 / *ApPGI1-1*	96.580	0.017070955	0.206122272	0.082819555	Purification selection
AtPGI1 / *ApPGI1-2*	96.585	0.016351402	0.185184129	0.088298072	Purification selection
AtHXK1 / *ApHXK1-1*	96.192	0.016110405	0.241283866	0.066769506	Purification selection
AtHXK1 / *ApHXK1-2*	95.795	0.017912636	0.204157582	0.087739264	Purification selection
AtIDD8 / *ApIDD8-1*	89.496	0.031409280	0.311738291	0.100755284	Purification selection
AtIDD8 / *ApIDD8-2*	89.006	0.035545868	0.343426052	0.103503702	Purification selection
AtADG1 / *ApADG1-1*	91.939	0.035091555	0.343030784	0.102298560	Purification selection
AtADG1 / *ApADG1-2*	91.747	0.038032815	0.344430181	0.110422424	Purification selection
AtPGM1 / *ApPGM1-1*	95.056	0.022179474	0.255276137	0.086884243	Purification selection
AtPGM1 / *ApPGM1-2*	95.056	0.021471555	0.261003181	0.082265490	Purification selection
AtTPS1 / *CsTPS1-1*	91.584	0.008492083	0.256483393	0.033109681	Purification selection
AtTPS1 / *CsTPS1-2*	90.981	0.010174782	0.252744167	0.040257236	Purification selection

AtTPS1 / *CsTPS1-3*	90.616	0.010209674	0.249271378	0.040958066	Purification selection
AtSNRK1.1 / *CsSNRK1.1-1*	98.441	0.005407608	0.211016385	0.025626485	Purification selection
AtSNRK1.1 / *CsSNRK1.1-2*	98.441	0.006264125	0.214912300	0.029147353	Purification selection
AtSNRK1.1 / *CsSNRK1.1-3*	98.428	0.007122392	0.234105159	0.030423898	Purification selection
AtPGI1 / *CsPGI1-1*	95.146	0.020770138	0.266728913	0.077869840	Purification selection
AtPGI1 / *CsPGI1-2*	94.805	0.023009900	0.281944598	0.081611424	Purification selection
AtPGI1 / *CsPGI1-3*	95.138	0.020778972	0.272702072	0.076196604	Purification selection
AtHXK1 / *CsHXK1-1*	95.372	0.024771448	0.298468499	0.082995183	Purification selection
AtHXK1 / *CsHXK1-2*	95.200	0.021124992	0.277648587	0.076085358	Purification selection
AtHXK1 / *CsHXK1-3*	95.000	0.022017456	0.278585585	0.079033007	Purification selection
AtIDD8 / *CsIDD8-1*	89.316	0.045943437	0.312769448	0.146892342	Purification selection
AtIDD8 / *CsIDD8-2*	89.362	0.045261267	0.356748483	0.126871645	Purification selection
AtADG1 / *CsADG1-1*	92.352	0.034242133	0.270302224	0.126680916	Purification selection
AtADG1 / *CsADG1-2*	91.238	0.038266396	0.287413777	0.133140439	Purification selection
AtADG1 / *CsADG1-3*	91.082	0.041795361	0.309945327	0.134847529	Purification selection
AtPGM1 / *CsPGM1-1*	93.770	0.026675886	0.261633355	0.101959043	Purification selection
AtPGM1 / *CsPGM1-2*	94.089	0.023837326	0.258881933	0.092077982	Purification selection
AtPGM1 / *CsPGM1-3*	93.610	0.026051823	0.271503172	0.095954029	Purification selection
AtTPS1 / *BnTPS1-1*	94.087	0.029508645	0.511502962	0.057690076	Purification selection
AtTPS1 / *BnTPS1-2*	93.559	0.025706022	0.475556168	0.054054649	Purification selection
AtTPS1 / *BnTPS1-3*	92.986	0.022313588	0.402708913	0.055408726	Purification selection
AtTPS1 / *BnTPS1-4*	90.262	0.020975365	0.413475221	0.050729437	Purification selection
AtSNRK1.1 / *BnSNRK1.1-1*	96.296	0.021060047	0.465442982	0.045247319	Purification selection
AtSNRK1.1 / *BnSNRK1.1-2*	96.296	0.020990410	0.465577393	0.045084685	Purification selection
AtSNRK1.1 / *BnSNRK1.1-3*	94.553	0.022495881	0.429194243	0.052414220	Purification selection
AtSNRK1.1 / *BnSNRK1.1-4*	94.163	0.023380402	0.419115704	0.055785079	Purification selection

AtPGI1 / *BnPGI1-1*	88.835	0.054501272	0.409567118	0.133070429	Purification selection
AtPGI1 / *BnPGI1-2*	90.610	0.037963946	0.47951917	0.079170862	Purification selection
AtPGI1 / *BnPGI1-3*	88.817	0.055939718	0.437028046	0.128000294	Purification selection
AtPGI1 / *BnPGI1-4*	89.786	0.045152666	0.464090537	0.097292796	Purification selection
AtHXK1 / *BnHXK1-1*	88.889	0.063668035	0.775598690	0.082088889	Purification selection
AtHXK1 / *BnHXK1-2*	89.113	0.063170901	0.734716061	0.085980019	Purification selection
AtHXK1 / *BnHXK1-3*	89.006	0.059354730	0.789800307	0.075151566	Purification selection
AtHXK1 / *BnHXK1-4*	89.006	0.061272677	0.806339797	0.075988656	Purification selection
AtIDD8 / *BnIDD8-1*	83.898	0.069187549	0.535738564	0.129144239	Purification selection
AtIDD8 / *BnIDD8-2*	83.263	0.068909307	0.516395270	0.133442947	Purification selection
AtADG1 / *BnADG1-2*	90.286	0.040269220	0.565200964	0.071247614	Purification selection
AtPGM1 / *BnPGM1-1*	91.209	0.032680215	0.270049062	0.121015842	Purification selection
AtTPS1 / *BoTPS1-1*	94.087	0.029508645	0.511502962	0.057690076	Purification selection
AtTPS1 / *BoTPS1-2*	94.474	0.022429819	0.389099133	0.057645513	Purification selection
AtSNRK1.1 / *BoSNRK1.1-1*	96.296	0.021060047	0.465442982	0.045247319	Purification selection
AtSNRK1.1 / *BoSNRK1.1-2*	92.898	0.023387196	0.418580664	0.055872613	Purification selection
AtSNRK1.1 / *BoSNRK1.1-3*	87.354	0.062715840	0.342335817	0.183199760	Purification selection
AtPGI1 / *BoPGI1-1*	84.653	0.060747164	0.396151692	0.153343190	Purification selection
AtHXK1 / *BoHXK1-1*	89.113	0.062197437	0.757847384	0.082071191	Purification selection
AtHXK1 / *BoHXK1-2*	89.006	0.061253807	0.815954500	0.075070125	Purification selection
AtIDD8 / *BoIDD8*	83.475	0.066819183	0.510542686	0.130878738	Purification selection
AtADG1 / *BoADG1-1*	91.188	0.043036288	0.593963011	0.072456176	Purification selection
AtPGM1 / *BoPGM*	92.540	0.030784565	0.258696710	0.118998671	Purification selection
AtTPS1 / *BrTPS1-1*	90.862	0.026189111	0.458301878	0.057143801	Purification selection
AtTPS1 / *BrTPS1-2*	90.967	0.025208384	0.395376097	0.063757986	Purification selection
AtSNRK1.1 / *BrSNRK1.1-1*	96.491	0.020409423	0.448233073	0.045533058	Purification selection

AtSNRK1.1 / *BrSNRK1.1-2*	94.553	0.022054012	0.426661883	0.051689670	Purification selection
AtSNRK1.1 / *BrSNRK1.1-3*	83.178	0.054675538	0.350843509	0.155840244	Purification selection
AtPGI1 / *BrPGI1-1*	88.817	0.055393667	0.428558370	0.129255829	Purification selection
AtPGI1 / *BrPGI1-2*	90.280	0.039502423	0.479011438	0.082466555	Purification selection
AtHXK1 / *BrHXK1-1*	89.091	0.062834942	0.791996084	0.079337441	Purification selection
AtHXK1 / *BrHXK1-2*	89.006	0.058404221	0.773309913	0.075524987	Purification selection
AtIDD8 / *BrIDD8*	81.568	0.070950392	0.536154259	0.132332050	Purification selection
AtADG1 / *BrADG1-1*	87.739	0.082251649	2.062148102	0.039886393	Purification selection
AtPGM1 / *BrPGM*	91.843	0.053214663	0.304380839	0.174829216	Purification selection
